# Combination COX-2 inhibitor and metformin attenuate rate of joint replacement in osteoarthritis with diabetes: A nationwide, retrospective, matched-cohort study in Taiwan

**DOI:** 10.1371/journal.pone.0191242

**Published:** 2018-01-31

**Authors:** Chieh-Hua Lu, Chi-Hsiang Chung, Chien-Hsing Lee, Chang-Hsun Hsieh, Yi-Jen Hung, Fu-Huang Lin, Chang-Huei Tsao, Po-Shiuan Hsieh, Wu-Chien Chien

**Affiliations:** 1 Department of Internal Medicine, Division of Endocrinology and Metabolism, Tri-Service General Hospital, School of Medicine, National Defense Medical Center, Taipei, Taiwan, ROC; 2 Department of Medical Research, National Defense Medical Center, Taipei, Taiwan, ROC; 3 School of Public Health, National Defense Medical Center, Taipei, Taiwan, ROC; 4 Taiwanese Injury Prevention and Safety Promotion Association, Taipei, Taiwan, ROC; 5 Department of Medical Research, Tri-Service General Hospital, National Defense Medical Center, Taipei, Taiwan, ROC; 6 Department of Microbiology & Immunology, National Defense Medical Center, Taipei, Taiwan, ROC; 7 Department of Physiology and Biophysics, National Defense Medical Center, Taipei, Taiwan, ROC; 8 Institute of Preventive Medicine, National Defense Medical Center, Taipei, Taiwan, ROC; Weill Cornell Medical College Qatar, QATAR

## Abstract

**Background:**

Osteoarthritis (OA) is the most common form of arthritis associated with an increased prevalence of type 2 diabetes mellitus (T2DM), however their impact on decreasing joint replacement surgery has yet to be elucidated. This study aimed to investigate if the combination of COX-2 inhibitor and metformin therapy in OA with T2DM were associated with lower the rate of joint replacement surgery than COX-2 inhibitor alone.

**Methods:**

In total, 968 subjects with OA and T2DM under COX-2 inhibitor and metformin therapy (case group) between 1 January to 31 December 2000 were selected from the National Health Insurance Research Database of Taiwan, along with 1936 patients were the 1:2 gender-, age-, and index year-controls matched without metformin therapy (control group) in this study. Cox proportional hazards analysis was used to compare the rate of receiving joint replacement surgery during 10 years of follow-up.

**Results:**

At the end of follow-up, 438 of all enrolled subjects (15.08%) had received the joint replacement surgery, including 124 in the case group (12.81%) and 314 in the control group (16.22%). The case group tended to be associated with lower rate of receiving the joint replacement surgery at the end of follow-up than the control group (p = 0.003). Cox proportional hazards regression (HR) analysis revealed that study subjects under combination therapy with metformin had lower rate of joint replacement surgery (adjusted HR 0.742 (95% CI = 0.601–0.915, p = 0.005)). In the subgroups, study subjects in the combination metformin therapy who were female, good adherence (>80%), lived in the highest urbanization levels of residence, treatment in the hospital center and lower monthly insurance premiums were associated with a lower risk of joint replacement surgery than those without.

**Conclusions:**

Patients who have OA and T2DM receiving combination COX-2 inhibitors and metformin therapy associated with lower joint replacement surgery rates than those without and this may be attributable to combination therapy much more decrease pro-inflammatory factors associated than those without metformin therapy.

## Introduction

Osteoarthritis(OA) is the most common form of arthritis and possesses marked variability of disease expression. The incidence of OA is rising because of the ageing population and the epidemic of obesity. [1] OA has a predilection for the hand, knee, hip, and spine, and less commonly affects the shoulder, elbow, wrist, and ankle. OA may be diagnosed without the use of radiography and/or laboratory investigations in the presence of typical symptoms and signs in the at-risk age group. [2] One recent hypothesis has suggested a new classification for phenotyping OA that includes ageing, metabolic syndrome(Mets) and post-traumatic events and genetic-related OA. [3]

OA is also associated with an increased prevalence of Mets studied in NHANES III data, [4] the other components of Mets, such as type 2 diabetes mellitus(T2DM), hypertension or dyslipidemia may cause OA pathophysiology. [5] The first paper describing an association between OA and diabetes was published in 1961. [6] Insulin resistance(IR) and T2DM seemed to be associated with OA in the Ulm OA and ROAD studies. [7, 8]http://rmdopen.bmj.com/content/1/1/e000077-ref-8 In addition, the link between the two diseases may be supported by the accumulation of advanced glycation end products, oxidative stress and promotion of systemic inflammation. [9, 10] Moreover, one recent meta-analysis highlights a high frequency of OA in patients with T2DM and an association between both diseases. [11]

The goals of OA management are to minimize pain, optimize function, and beneficially modify the process of joint damage. Pain and loss of function are the main clinical features that lead to treatment, including biomechanical interventions, exercise (land-based and water-based), self-management and education, strength training, and weight management, pharmacological, and surgical approaches. [12, 13] Clinical trial data show that the traditional nonsteroidal anti-inflammatory drugs (NSAIDs) are more effective than acetaminophen in the treatment of patients with symptoms and signs of OA. [14, 15] In patients with comorbidities such as T2DM, hypertension, previous gastrointestinal bleeding and advanced age, a cyclooxygenase (COX)-2 selective NSAID should be better than NSAIDs. One meta-analysis enhanced that OA treatment with celecoxib was significantly improved than that with placebo. [16] However, surgical treatment is dominated in patients with advanced knee and hip OA when conservative therapies have failed to provide adequate pain relief. [17, 18]

Metformin is the preferred initial pharmacologic agent for the treatment of T2DM [19] that has been shown to reduce chronic inflammation indirectly through reduction of hyperglycemia, or directly acting as anti-inflammatory drug. [20] As described in detail previously that additional effect of metformin and celecoxib against adipose tissue inflammation that resulted in a reduction in adipose tissue macrophage infiltration and decreases in levels of adipose tissue TNF-α, MCP-1, and leptin levels in high-fat fed rats. [21] No previous study reported combination metformin and COX-2 inhibitor therapy in OA comorbid with T2DM associated with lower joint replacement surgery rate than used COX-2 inhibitor only. Therefore, the aim of this study was to clarify this association using data from a nationwide health insurance database, the Taiwan National Health Insurance Research Database (NHIRD).

## Materials and methods

### Data sources

In this study, we used data from the NHIRD to investigate combination metformin and COX-2 inhibitor therapy in OA comorbid with T2DM could lower joint replacement surgery rate than used COX-2 inhibitor only over a 10-year period, from the outpatient Longitudinal Health Insurance Database (LHID) in Taiwan (2000–2010). As described in detail previously, [22] the National Health Insurance (NHI) Program was launched in Taiwan in 1995, and as of June 2009 it included contracts with 97% of the medical providers in Taiwan with approximately 23 million beneficiaries, or more than 99% of the entire population in Taiwan. [23] The NHIRD uses International Classification of Diseases, 9th Revision, Clinical Modification (ICD-9-CM) codes to record diagnoses. [24] All diagnoses of T2DM were made by board-certified medical specialist, and OA were confirmed by orthopedic specialist. The Bureau of NHI randomly reviews the records of 1 in 100 ambulatory care visits and 1 in 20 in-patient claims to verify the accuracy of the diagnoses. [25] Several studies have demonstrated the accuracy and validity of the diagnoses in the NHIRD. [26, 27]

### Study design and sampled participants

**Fig 1:**
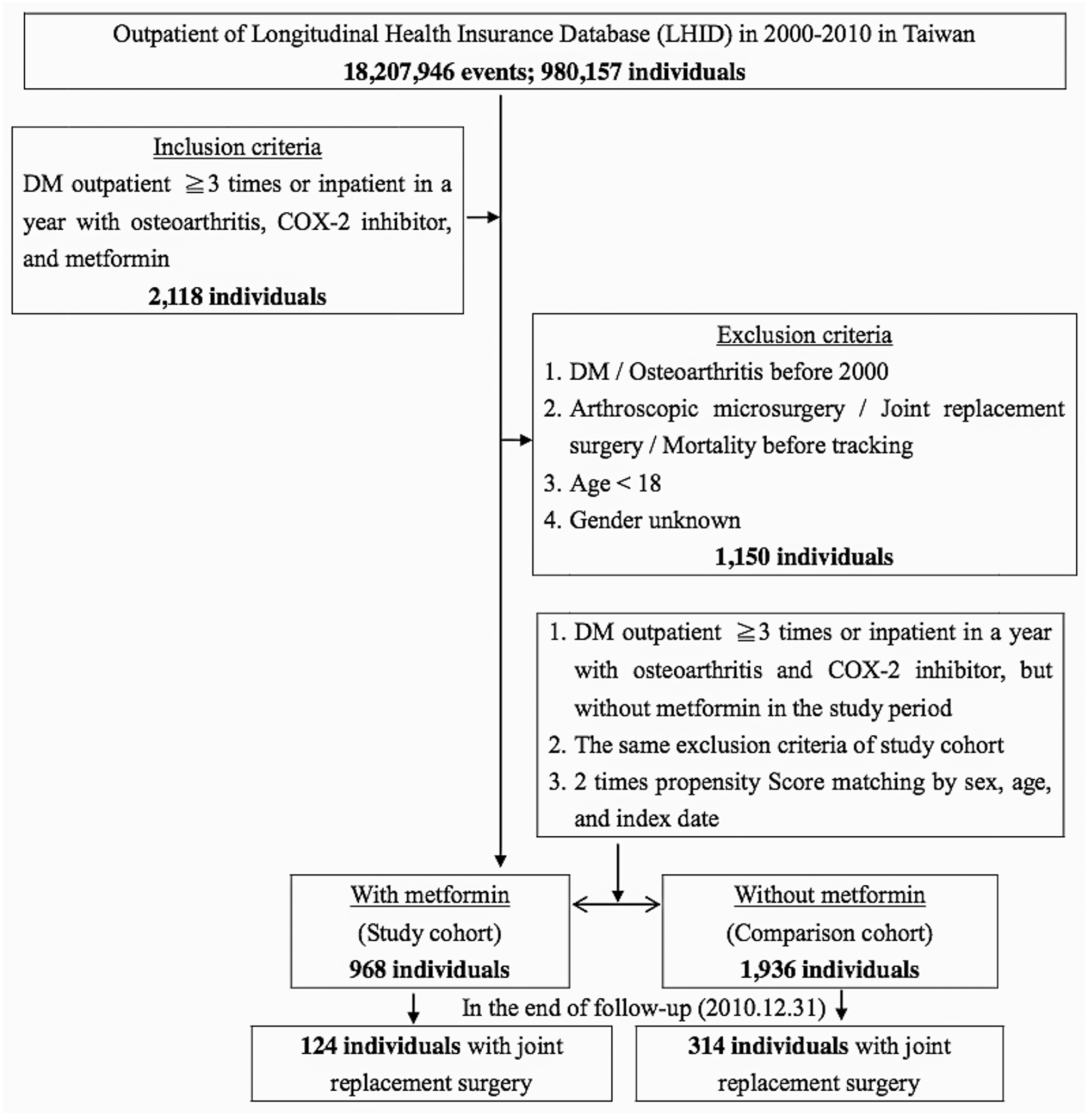
The flowchart of study sample selection from National Health Insurance Research Database in Taiwan. DM = Diabetes mellitus: ICD-9-CM 250; Osteoarthritis: ICD-9-CM 715; COX-2 inhibitor / Metformin: ≧ 90 days. Arthroscopic microsurgery was including synovectomy or/and capsulotomy (NHIRD order code 64054B-64057B), partial meniscectomy (NHIRD order code 64218B), and arthroscopic surgery (NHIRD order code 64243B-64244B). Joint replacement surgery was including osteosynthesis (NHIRD order code 64038B-64040B), total hip replacement (NHIRD order code 64162B-64168B), partial joint replacement (NHIRD order code 64169B-64170B), arthroplasty (NHIRD order code 64171B-64177B), arthrodesis (NHIRD order code 64178B-64183B), removal of prosthesis (NHIRD order code 64198B-64200B), revision replacement (64201B-64202B), and girdle stone procedure of hip (NHIRD order code 64203B).

This study was a retrospective matched-cohort design. Patients with diagnosed OA and T2DM were selected from 1 January 2000 to 31 December 2010 according to ICD-9-CM 715.XX (OA) and ICD-9-CM 250.XX (T2DM). In addition, each enrolled patient was required to have made at least 3 outpatient visits within the study period according to these ICD-9-CM codes under COX-2 inhibitors therapy with or without metformin therapy. The patients with OA and/or T2DM before 2000 were excluded. In addition, the patients received joint replacement surgery before tracking were also excluded. All patients aged <18 years were also excluded. A total of 2118 enrolled patients that excluded 1150 patients then the 968 subjects with OA and T2DM under COX-2 inhibitor and metformin therapy (case group) along with 1936 patients were the 1:2 sex-, age-, and index year-controls matched without metformin therapy (control group) in this study. (Fig 1)

The covariates included gender, age, Charlson Comorbidity Index(CCI) removed T2DM, geographical area of residence (north, center, south, east of Taiwan, and outlets islands), urbanization level of residence (level 1 the highest; level 3 the lowest) and monthly income (in New Taiwan Dollars [NTD]; <10,000, 10,000–14,999, ≥15,000). The urbanization level of residence was defined according to the population and various indicators of the level of development. Level 1 was defined as a population >1,250,000, and a specific designation as political, economic, cultural and metropolitan development. Level 2 was defined as a population between 500,000 and 1249,999, and as playing an important role in the political system, economy, and culture. Urbanization levels 3 was defined as a population <500,000. [28]

### Outcome measures

All of the study participants were followed from the index date until the onset of receiving joint replacement surgery from the NHI program before the end of 2010.

### Statistical analysis

All analyses were performed using SPSS software version 22 (SPSS Inc., Chicago, Illinois, USA). Conditional logistic regression was used to evaluate the distributions of study and control groups. Multivariable Cox proportional hazards regression analysis was used to determine the risk of receiving joint surgical replacement, and the results were present as adjusted hazard ratio with 95% confidence interval (CI). The difference in the risk of receiving joint surgical replacement between the study and control groups was estimated using the Kaplan-Meier method with the log-rank test. A 2-tailed p value <0.05 was considered to indicate statistical significance.

### Ethics

This study was conducted in accordance with the Code of Ethics of the World Medical Association (Declaration of Helsinki). The Institutional Review Board of Tri-Service General Hospital approved this study and waived the need for individual written informed consent (TSGH IRB No. 2-105-05-082).

## Results

Table 1 shows the gender, age, comorbidities, location, urbanization, level of care and income of the study subjects and controls. Compared to the controls, the study subjects much more tended to receive therapy in hospital center and have more lived in higher urbanized areas, northern areas of Taiwan (p < 0.001).

**Table 1:** Characteristics of study in the baseline.

Metformin	Total		With		Without		*P*
Variables	n	*%*	n	*%*	n	*%*	
**Total**	2,904		968	*33*.*33*	1,936	*66*.*67*	
**Gender**							
Male	1,245	*42*.*87*	415	*42*.*87*	830	*42*.*87*	Reference
Female	1,659	*57*.*13*	553	*57*.*13*	1,106	*57*.*13*	0.852
**Age (years)**	70.55±10.66		70.17±10.85		70.74±10.56		0.070
**CCI removed DM**	0.71±1.16		0.80±1.26		0.67±1.10		0.008
**Location**							
Northern Taiwan	1,013	*34*.*88*	439	*45*.*35*	574	*29*.*65*	Reference
Middle Taiwan	867	*29*.*86*	204	*21*.*07*	663	*34*.*25*	<0.001
Southern Taiwan	775	*26*.*69*	244	*25*.*21*	531	*27*.*43*	<0.001
Eastern Taiwan	224	*7*.*71*	74	*7*.*64*	150	*7*.*75*	0.378
Outlets islands	25	*0*.*86*	7	*0*.*72*	18	*0*.*93*	0.589
**Urbanization level**							
1 (The highest)	817	*28*.*13*	362	*37*.*40*	455	*23*.*50*	<0.001
2	1,187	*40*.*87*	393	*40*.*60*	794	*41*.*01*	0.011
3 (The lowest)	900	*40*.*00*	213	*22*.*00*	687	*35*.*49*	Reference
**Level of care**							
Hospital center	925	*31*.*85*	368	*38*.*02*	557	28.77	0.274
Regional hospital	1,164	*40*.*08*	367	*37*.*91*	797	*41*.*17*	0.035
Local hospital	815	*28*.*06*	233	*24*.*07*	582	*30*.*06*	Reference
**Insured premium (NT$)**							
<10,000	1,469	*50*.*58*	488	*50*.*41*	981	*50*.*67*	Reference
10,000–14,999	730	*25*.*14*	251	*25*.*93*	479	*24*.*74*	0.375
≧15,000	705	*24*.*28*	229	*23*.*66*	476	*24*.*59*	0.362

CCI = Charlson Comorbidity Index

*P*: Conditional logistic regression

Table 2 shows that at the end of follow-up, 438 of all enrolled subjects (15.08%) had received the joint replacement surgery, including 124 in the case group (12.81%) and 314 in the control group (16.22%). The case group tended to be associated with a lower rate of receiving the joint replacement surgery at the end of follow-up than the control group (p = 0.003).

**Table 2:** Characteristics of study in the endpoint.

Metformin	Total		With		Without		*P*
Variables	n	*%*	n	*%*	n	*%*	
**Total**	2,904		968	*33*.*33*	1,936	*66*.*67*	
**Joint replacement surgery**							
Without	2,466	*84*.*92*	844	*87*.*19*	1,622	*83*.*78*	Reference
With	438	*15*.*08*	124	*12*.*81*	314	*16*.*22*	0.003
**Gender**							
Male	1,245	*42*.*87*	415	*42*.*87*	830	*42*.*87*	0.653
Female	1,659	*57*.*13*	553	*57*.*13*	1,106	*57*.*13*	Reference
**Age (years)**	72.32±10.68		71.90±10.86		72.53±10.59		0.066
**CCI removed DM**	1.10±1.99		1.19±1.94		1.06±2.02		0.183
**Location**							
Northern Taiwan	1,021	*35*.*16*	429	*44*.*32*	592	*30*.*58*	Reference
Middle Taiwan	856	*29*.*48*	206	*21*.*28*	650	*33*.*57*	<0.001
Southern Taiwan	771	*26*.*55*	244	*25*.*21*	527	*27*.*22*	<0.001
Eastern Taiwan	227	*7*.*82*	80	*8*.*26*	147	*7*.*59*	0.229
Outlets islands	29	*1*.*00*	9	*0*.*93*	20	*1*.*03*	0.369
**Urbanization level**							
1 (The highest)	825	*28*.*41*	331	*34*.*19*	494	*25*.*52*	0.020
2	1,210	*41*.*67*	402	*41*.*53*	808	*41*.*74*	0.034
3 (The lowest)	869	*29*.*92*	235	*24*.*28*	634	*32*.*74*	Reference
**Level of care**							
Hospital center	922	*31*.*75*	338	*34*.*92*	584	*30*.*17*	0.393
Regional hospital	1,187	*40*.*87*	374	*38*.*64*	813	*41*.*99*	0.419
Local hospital	795	*27*.*38*	256	*26*.*45*	539	*27*.*84*	Reference
**Insured premium (NT$)**							
<10,000	1,469	*50*.*58*	488	*50*.*41*	981	*50*.*67*	Reference
10,000–14,999	730	*25*.*14*	251	*25*.*93*	479	*24*.*74*	0.375
≧15,000	705	*24*.*28*	229	*23*.*66*	476	*24*.*59*	0.362

CCI = Charlson Comorbidity Index

*P*: Conditional logistic regression

**Fig 2:**
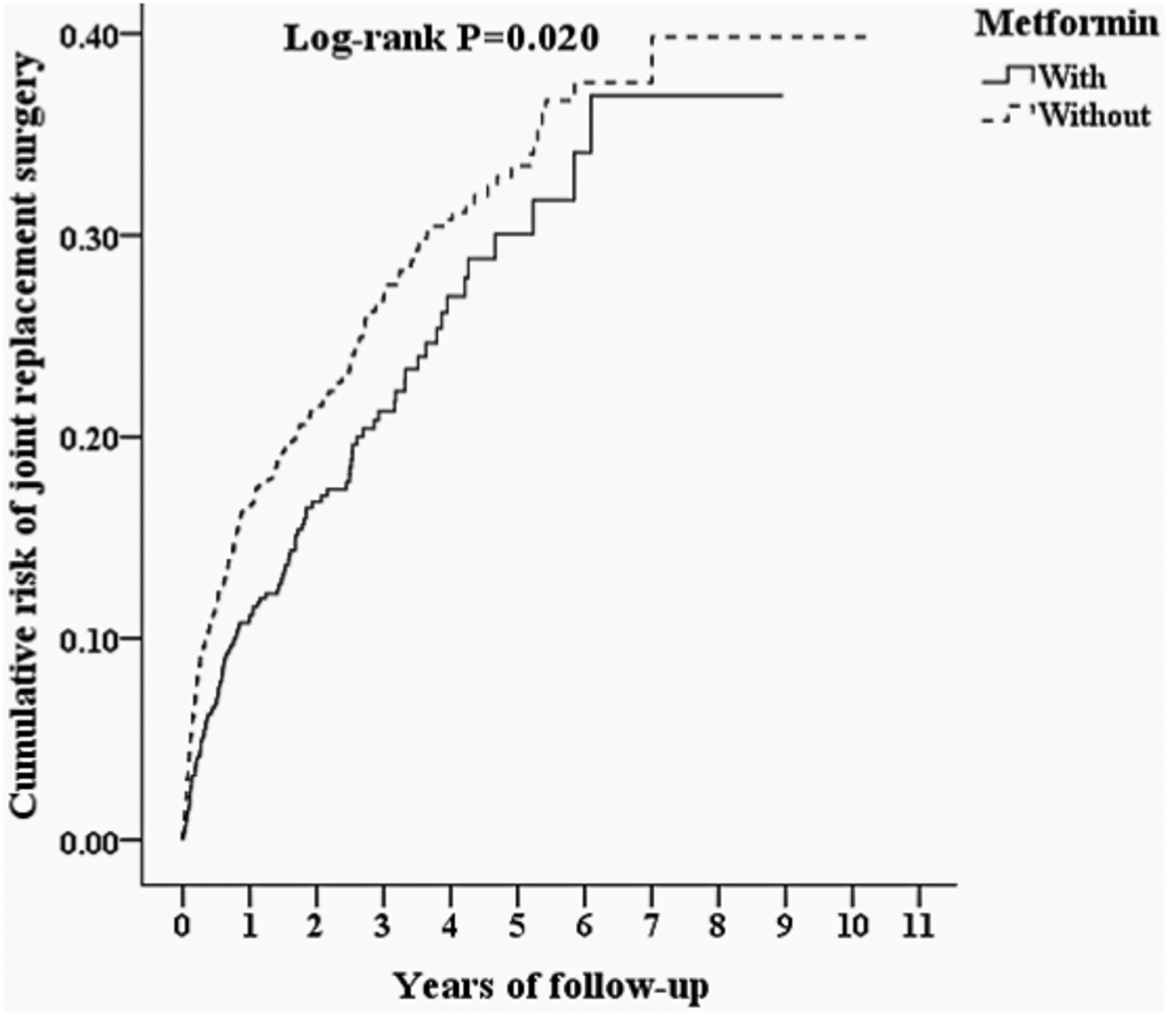
Kaplan-Meier for cumulative risk of joint replacement surgery among DM with osteoarthritis and COX-2 inhibitor patients aged 18 and over stratified by metformin with log-rank test.

Fig 2 shows the Kaplan-Meier analysis for the cumulative risk of joint replacement surgery in the case and control groups with difference statistically significant (log-rank, p = 0.02). In addition, at the first year of follow-up, the difference between the two groups became significant (log-rank test <0.001).

Table 3 shows the results of Cox regression analysis of the factors associated with the rate of joint replacement surgery. Cox proportional hazards regression (HR) analysis revealed that study subjects under metformin therapy were associated with lower rate of joint replacement surgery (adjusted HR 0.742 (95% CI = 0.601–0.915, p = 0.005)). The study subjects with increasing aged, medication possession ratio (MPR) with good adherence (>80%), high comorbidity (CCI scores) who removed diabetic patients and lived in non-Northern areas of Taiwan were associated with lower rate receiving joint replacement surgery. Otherwise, the study subjects lived in higher urbanized areas and receive therapy in hospital center were associated with higher rate receiving joint replacement surgery.

**Table 3:** Factors of joint replacement surgery by using Cox regression.

Variables	Crude HR	95% CI Lower limit	95% CI Upper limit	*P*	Adjusted HR	95% CI Lower limit	95% CI Upper limit	*P*
**Metformin**								
Without	Reference				Reference			
With	0.781	0.634	0.962	0.020	0.742	0.601	0.915	0.005
**MPR of metformin**								
Without	Reference							
<40%	0.885	0.629	1.162	0.316				
40–80%	0.797	0.598	1.061	0.120				
>80%	0.634	0.412	0.977	0.039				
**Gender**								
Male	0.850	0.701	1.029	0.095	0.920	0.757	1.117	0.399
Female	Reference				Reference			
**Age (years)**	0.969	0.962	0.977	<0.001	0.971	0.964	0.979	<0.001
**CCI removed DM**	0.612	0.544	0.688	<0.001	0.647	0.578	0.725	<0.001
**Location**					**Had collinearity with urbanization level**			
Northern Taiwan	Reference							
Middle Taiwan	0.613	0.484	0.775	<0.001				
Southern Taiwan	0.705	0.555	0.895	0.004				
Eastern Taiwan	0.628	0.429	0.918	0.016				
Outlets islands	0.817	0.303	2.200	0.689				
**Urbanization level**								
1 (The highest)	1.773	1.452	2.457	0.006	1.734	1.401	2.001	0.013
2	1.245	1.126	2.118	0.012	1.201	1.096	1.978	0.027
3 (The lowest)	Reference				Reference			
**Level of care**								
Hospital center	1.995	1.554	2.561	<0.001	1.673	1.265	2.214	<0.001
Regional hospital	1.258	0.978	1.618	0.074	1.113	0.862	1.437	0.410
Local hospital	Reference				Reference			
**Insured premium (NT$)**								
<10,000	Reference				Reference			
10,000–14,999	1.459	0.875	3.742	0.305	1.269	0.855	3.307	0.256
≧15,000	1.546	0.903	3.886	0.496	1.334	0.894	3.521	0.452

HR = hazard ratio; CI = confidence interval; Adjusted HR: Adjusted variables listed in the table

In the subgroups stratified by the gender, urbanization, level of care and monthly income, the study subjects who were female, lived in the highest urbanization levels of residence, treatment in the hospital center and monthly insurance premiums of NT$ <10,000 were associated with a lower risk of joint replacement surgery in the combination metformin therapy group than those without metformin which respectively adjusted HR as 0.687 (p = 0.008), 0.549 (p = 0.003), 0.625 (p = 0.005), and 0.740 (p = 0.003) (Table 4). The patients stratified by MPR, in those with good adherence (>80%) with a trend that associated lower adjusted HR 0.566 (95% CI = 0.366–0.875, p = 0.01) (Table 5).

**Table 4:** Factors of joint replacement surgery stratified by variables listed in the table by using Cox regression.

Metformin	With			Without			Ratio	Adjusted HR	95%CI Lower limit	95%CI Upper limit	*P*
Stratified	Event	PYs	Rate (per 10^5^ PYs)	Event	PYs	Rate (per 10^5^ PYs)					
**Total**	124	5,014.97	2,472.60	314	10,402.46	3,018.52	0.819	0.742	0.601	0.915	0.005
**Gender**											
Male	53	2,179.67	2,431.56	121	4,404.22	2,747.37	0.885	0.855	0.616	1.189	0.352
Female	71	2,835.30	2,504.14	193	5,998.24	3,217.61	0.778	0.687	0.522	0.905	0.008
**Urbanization level**											
1 (The highest)	37	1,449.69	2,552.27	96	2,294.72	4,183.52	0.610	0.549	0.371	0.811	0.003
2	66	2,255.01	2,926.82	140	4,402.77	3,179.82	0.920	0.881	0.656	1.184	0.401
3 (The lowest)	21	1,310.27	1,602.72	78	3,704.97	2,105.28	0.761	0.668	0.454	1.752	0.164
**Level of care**											
Hospital center	49	1,535.77	3,190.58	127	2,580.49	4,921.55	0.648	0.625	0.449	0.871	0.005
Regional hospital	44	1,971.72	2,231.55	123	4,569.93	2,691.51	0.829	0.809	0.567	1.153	0.241
Local hospital	31	1,507.48	2,056.41	64	3,252.04	1,968.00	1.045	0.920	0.695	1.421	0.706
**Insured premium (NT$)**											
<10,000	113	2,511.24	4,499.77	292	5,301.70	5,507.67	0.817	0.740	0.599	0.913	0.003
10,000–14,999	7	1,413.39	495.26	13	2,577.24	504.42	0.982	0.889	0.720	1.097	0.129
≧15,000	4	1,090.34	366.86	9	2,523.52	356.64	1.029	0.932	0.755	1.149	0.337

PYs = Person-years; Adjusted HR = Adjusted Hazard ratio: Adjusted for the variables listed in Table 3.; CI = confidence interval

**Table 5:** Factors of joint replacement surgery stratified by MPR of metformin by using Cox regression.

Metformin	With			Without			Ratio	Adjusted HR	95%CI Lower limit	95%CI Upper limit	*P*
MPR	Event	PYs	Rate (per 10^5^ PYs)	Event	PYs	Rate (per 10^5^ PYs)					
**Total**	124	5,014.97	2,472.60	314	10,402.46	3,018.52	0.819	0.742	0.601	0.915	0.005
<40%	47	1,700.27	2,764.27				0.916	0.828	0.608	1.126	0.916
40–80%	55	2,150.56	2,557.47				0.847	0.757	0.567	1.009	0.847
>80%	22	1,164.14	1,889.81				0.626	0.566	0.366	0.875	0.626

PYs = Person-years; Adjusted HR = Adjusted Hazard ratio: Adjusted for the variables listed in Table 3.; CI = confidence interval

## Discussion

Previous studies have reported that a high frequency of OA in patients with T2DM and COX-2 inhibitors were significantly improved joint pain. [11, 16] Furthermore, some studies addressing the influence of T2DM on OA and its therapeutic outcomes suggests that DM may augment the development and severity of OA and that T2DM increases risks associated with joint replacement surgery. However, no studies discussed that combination COX-2 inhibitors and metformin therapy in OA patients with T2DM were associated with lower joint replacement surgery rates.

We found that the OA patients with T2DM under COX-2 inhibitors and metformin therapy were associated with lower joint replacement surgery rates than COX-2 inhibitors only. Even after adjusting for comorbidities and other covariates, the over-all adjusted HR was 0.742 (95% CI 0.601–0.915, P = 0.005). Kaplan-Meier analysis revealed that the study subjects were associated with a significantly lower 10-year risk of joint replacement surgery than the controls. In addition, it took just only 1 year to achieve a significantly adjusted HR. Our study is the first to indicate that OA patients with T2DM under COX-2 inhibitors combined metformin therapy were associated with lower joint replacement surgery risk in a nationwide, population-based study.

OA is a heterogeneous disorder that metabolic OA is wider than obesity-related OA since metabolic syndrome and OA are epidemiologically linked. [4, 12] In addition, one study showed that OA and T2DM were significantly associated that overall risk of OA in the T2DM population was 1.46 (1.08 to 1.96) and that of T2DM in the OA population was 1.41 (1.21 to 1.65). [11] Moreover, previous studies showed that T2DM independently alters the prognosis by increasing the risk of total joint replacement [29] and could be a specific OA risk factor. [30, 31] Long-standing T2DM is independently associated with advanced OA of knee and hip joints. The mechanisms responsible for the T2DM is a predictor for severe OA remain unclear. Schett et al have shown that impact of T2DM on symptoms or on OA structural lesions were more severe in T2DM than those without T2DM. [29] Moreover, the inflammatory aspect in imaging corroborates with the higher release of inflammatory mediators in OA cartilage explants from T2DM than those without T2DM. [32]

This finding adds to the yet short list of risk predictors for OA established in prospective evaluations. [33–35] After controlling the analysis for age, BMI, and other potential confounders, T2DM comprised a twofold risk of severe OA necessitating arthroplasty. These data suggest that hypertension, hypercholesterolemia, and blood glucose are associated with both unilateral and bilateral knee OA independent of obesity, and support the concept that OA has an important systemic and metabolic component in its etiology.

High glucose in T2DM may participate in IL-1β–induced inflammation via oxidative stress and the polyol pathway that increased inflammation in OA. [32] Much more increased IL-1β–induced IL-6 and PGE2 production in OA cartilage from T2DM than non-diabetes group which associated with IL-6 and COX2 mRNA expression, IL-6 and PGE2 release, and ROS and NO production in cultured chondrocytes. The traditional NSAIDs or selective COX-2 inhibitors are effective in the treatment of patients with symptoms and signs of OA. [14–16] When conservative treatment options are limited, and surgical replacement of damaged joints may be necessary in severe OA patients.

Our previous study showed that additional effect of metformin and celecoxib against lipid dysregulation and adipose tissue inflammation in high-fat fed rats with IR and fatty liver. [21] Combination therapy with celecoxib and metformin resulted in a reduction in adipose tissue macrophage infiltration and decreases in levels of adipose tissue TNF-α, MCP-1, and leptin levels in high-fat fed rats. We therefore hypothesize that combination therapy with COX-2 inhibitors and metformin may decrease these inflammatory factors resulting in OA patients with T2DM were associated with lower joint replacement surgery rates than COX-2 inhibitors only. It may be associated with additional effect of combination therapy in against inflammatory factors of OA that lower joint replacement surgery rates.

In our study, the subjects shows the factors associated with lower rate of joint replacement surgery stratified by MPR in those with good adherence. Treatment adherence is critical to effective management of T2DM or other chronic systemic diseases. [36, 37] Patient with well treatment adherence is important that poor adherence contributes to disease progression and increased morbidity and mortality. [38] In Taiwan, the average life expectancy is 76.0 years in males and 82.5 years in females, [39] and it is important for the elderly with much higher CCI with much more risk for anesthesia and surgery then caused a lower rate for joint replacement surgery. However, the reasons why the subgroups lived in higher urbanized areas, received therapy in hospital center, lower monthly insurance premiums and the female patients associated with a lower rate for joint replacement surgery are unknown, and further studies are needed to clarify this issue.

### Limitations

There are several limitations to this study. First, patients with OA or T2DM could be identified using the insurance claims data, however data on the severity, disease duration and impact on diabetes control as HbA1c level were not available. Second, medical treatment may be effective in symptom improvement by decreasing inflammatory factors, however details regarding OA assessment scores were not available in the NHIRD. Finally, a longer follow-up period may be necessary to clarify risk for patients receiving joint replacement surgery.

## Conclusion

Patients with T2DM are at a higher risk of developing OA than those without T2DM. Combination COX-2 inhibitors and metformin therapy in OA patients with T2DM are associated with lower joint replacement surgery rates than COX-2 inhibitor alone. Although the mechanisms responsible for this association are still unclear, inflammatory factors may contribute to the decreasing surgical rate. Further studies on the effects in reducing the joint replacement surgery risk of OA with T2DM are warranted.
